# Highlighting Protective Effect of Encapsulation on Yeast Cell Response to Dehydration Using Synchrotron Infrared Microspectroscopy at the Single-Cell Level

**DOI:** 10.3389/fmicb.2020.01887

**Published:** 2020-08-07

**Authors:** Thanh Dat Nguyen, Stéphane Guyot, Caroline Pénicaud, Stéphanie Passot, Christophe Sandt, Fernanda Fonseca, Rémi Saurel, Florence Husson

**Affiliations:** ^1^UMR PAM A 02.102, AgroSup Dijon, Université Bourgogne Franche-Comté, Dijon, France; ^2^INRAE, AgroParisTech, Université Paris-Saclay, Thiverval-Grignon, France; ^3^SMIS Beamline, Synchrotron Soleil, Saint-Aubin, France

**Keywords:** yeast, encapsulation, dehydration, response, synchrotron FTIR

## Abstract

In the present paper, the Layer by Layer (LbL) method using β-lactoglobulin and sodium alginate was performed to individually encapsulate *Saccharomyces cerevisiae* cells in microorganized shells in order to protect them against stresses during dehydration. Higher survival (∼1 log) for encapsulated yeast cells was effectively observed after air dehydration at 45°C. For the first time, the potentiality of Synchrotron-Fourier Transform InfraRed microspectroscopy (S-FTIR) was used at the single-cell level in order to analyze the contribution of the biochemical composition of non-encapsulated vs. encapsulated cells in response to dehydration. The microspectroscopy measurements clearly differentiated between non-encapsulated and encapsulated yeast cells in the amide band region. In the spectral region specific to lipids, the S-FTIR results indicated probably the decrease in membrane fluidity of yeast after dehydration without significant distinction between the two samples. These data suggested minor apparent chemical changes in cell attributable to the LbL system upon dehydration. More insights are expected regarding the lower mortality among encapsulated cells. Indeed the hypothesis that the biopolymeric layers could induce less damage in cell by affecting the transfer kinetics during dehydration-rehydration cycle, should be verified in further work.

## Introduction

*Saccharomyces cerevisiae* is an eukaryotic model organism widely used in many technological applications in food industries and in biofuel production. Before use, yeast end-production requires long-term stabilization processes preserving the viability of cells and maintaining their functionality. The main method used to preserve yeast is dehydration. However, during this process, cells are subjected to several stresses such as mechanical, hydric, thermal and oxidative stresses, and inducing cell death (Beker and Rapoport, 1987; Fu and Chen, 2011). Many previous studies have identified the plasma membrane as one of the main targets of cell injury associated to hydric disturbance in cell environment (Guyot et al., 2006; Simonin et al., 2007; Dupont et al., 2011; Penicaud et al., 2014; Câmara et al., 2019). In particular, in our previous study (Nguyen et al., 2017), synchrotron infrared spectroscopy revealed biochemical modifications during dehydration/rehydration processes of *S. cerevisiae* related to membrane fluidity. The cell wall, cytoplasmic membrane, nucleoplasm, and vacuoles may be irreversibly destroyed during dehydration, as reported elsewhere (Beker and Rapoport, 1987). On the other hand, dehydration in aerobic conditions increases the contact between cell surfaces and air, thus inducing the accumulation of reactive oxygen species (Gamero-Sandemetrio et al., 2014). Another previous works have shown that cell oxidation level increased significantly during air-drying (Pereira de Jusus et al., 2003; Garre et al., 2010). Oxidation phenomena cause damage to cell proteins, lipids, and nucleic acids (França et al., 2007) with severe consequences on the overall metabolism (Hansen et al., 2006).

Otherwise, yeast survival can be improved by using encapsulation methods. Among microencapsulation methods, the layer-by-layer (LbL) self-assembly technique have demonstrated particular interest to control cell viability and functionality (Kiprono et al., 2018). The LbL system is built by means of electrostatic attraction between oppositely charged polyelectrolytes forming successive layers deposited around the active core (Guzmán et al., 2017). This technique offers several advantages such as control over the required multilayer thickness (the wall thickness of capsules can be tailored in the nm-μm range by adjusting the counter-ions, polyelectrolyte concentration, and pH), a broad selection of shell materials among several types of synthetic/natural charged colloids, the simplicity of the LbL process and equipment, and good biocompatibility and biodegradability of most of the polyelectrolytes (de Villiers et al., 2011; Guzmán et al., 2017). Moreover, the shell produced by this method is able to enhance cell resistance to external stresses such as chemical stress, heating, malnutrition or enzymatic action, and to control cell division (Hong et al., 2013; Anselmo et al., 2016). Recently, the LbL encapsulation method was successfully applied to *S. cerevisiae* cells by our group and others (Nguyen et al., 2015; Kim et al., 2016; Nguyen, 2016; Moon et al., 2020). In our previous work (Nguyen et al., 2015), the protection of yeast cells against freezing and some chemical stresses by using a multilayer system based on β-lactoglobulin and alginate has been evidenced. In this refered work, Fourier-transform infrared (FTIR) spectroscopy was used to demonstrate that the LbL biopolymeric system did not affect the cell wall structure and preserve cell functionality.

Fourier-transform infrared spectroscopy is a powerful technique that reveals information on functional groups or bonds in biochemical components such as proteins, lipids, nucleic acids, and carbohydrates of the whole cell, cell wall and membranes. Compared to classical FTIR spectroscopy systems, synchrotron radiation infrared beam source offers 100–1000 times higher brightness than that of a Globar IR source (Miller and Dumas, 2006). This powerful technique is crucial to examine very small samples, opening up investigation of biochemical composition at the single-cell level and observation of the heterogeneity of cellular behavior affected by surrounding conditions. The potential of synchrotron FTIR (S-FTIR) microspectroscopy for imaging and spatially resolving biochemical analyses at the single-cell scale was demonstrated in previous works. Saulou et al. (2010, 2013) assessed the changes in *S. cerevisiae* and *Escherichia coli* after exposure to nano-silver coating and free ionic silver, respectively. By simultaneously acquiring information on the biochemical and physiological properties of *Lactobacillus delbrueckii* cells, Passot et al. (2015) were able to assess relevant spectral biomarkers of the cryotolerance of lactic acid bacteria. Moreover, this technique provides an opportunity to examine physiological and biochemical changes in yeast cells during dehydration in order to identify more precisely the molecular biomarkers associated to stress (Nguyen et al., 2015; Câmara et al., 2019).

In the present study, *S. cerevisiae* yeast cells were individually encapsulated by using the same LbL method as proposed in our previous work (Nguyen et al., 2015) in order to improve cell survival during a dehydration process. The protective effect of the biopolymeric capsule using β-lactoglobulin and sodium alginate as natural polyelectrolytes, was evaluated by means of cell survival measurements. In addition, the potential of S-FTIR method was evaluated in terms of analyzing the biochemical modifications of *S. cerevisiae* upon dehydration at the single-cell level.

## Experimental

### Yeast Strain and Materials

The yeast strain *S. cerevisiae* BY4742 (*MAT*α *his3*Δ*1 leu*Δ*0 lys2*Δ*0 ura3*Δ*0*) was provided by Euroscarf (Frankfurt, Germany). Glucose, potassium acetate, sodium acetate, purified β-lactoglobulin (βlg, >90% purity), and L-cystein were all obtained from Sigma–Aldrich (Saint-Quentin Fallavier, France). Yeast extract and Agar were obtained from AES laboratory (Bruz, France). Peptone was obtained from Biokar diagnostics (Beauvais, France). Potassium dihydrogen phosphate (KH_2_PO_4_) was obtained from Prolabo (Leuven, Belgium). Sodium alginate (Alg) was obtained from Fisher Scientific (Strasbourg, France). Alg was used after purification and freeze-drying as indicated in our previous paper (Nguyen et al., 2015). The intrinsic viscosity (η) of alginate measured by capillary viscosimetry in 0.1 M NaCl at 20°C before and after purification was 554 and 485 mL/g, respectively.

### Culture Conditions

Stored yeast cells were grown with an initial OD_600_ of 0.2 in 50 mL of a medium containing 30 g/L Glucose, 30 g/L Yeast extract, 0.6 g/L KH_2_PO_4_, and 0.6 g/L L-Cysteine for pre-culture and culture. It was prepared in 250 mL battled Erlenmeyer flasks and shaken in an incubator at 140 rpm and 30°C for 24 h. The cells in the stationary phase were recovered by centrifugation, and were then washed twice with acetate buffer at pH 3.8 before use. All microbiological experiments were carried out in triplicate from independent pre-cultures.

### Encapsulation Procedure

Yeast cells were encapsulated as described in our previous study (Nguyen et al., 2015). The cells were suspended in acetate buffer at pH 3.8. The β-lactoglobulin (βlg) and sodium alginate (Alg) solutions were prepared at 0.1% (v/v) and 0.01% (v/v) concentrations, respectively. The cells were first incubated with the βlg solution, then the Alg solution, and finally the βlg solution. It means that the cells were encapsulated with three layers of biopolymers as it has been previously evidenced (Nguyen et al., 2015). In this previous work, the thickness of the three layers was evaluated in the range of 140 to 190 nm. The cell suspension at a final cellular concentration of 4.10^7^ cells/mL was maintained under low stirring for 20 min at room temperature for complete adsorption. The polyelectrolytes which were not adsorbed were removed by washing twice with an acetate buffer between steps. At each step, the yeast cells were separated from the supernatant by centrifugation at 2000 × *g* for 3 min.

### Dehydration Process

Yeast cells were dehydrated at 45°C following a previously described process (Lemetais et al., 2012). The specific dehydration chamber consisted of an airtight plastic box with controlled relative humidity. A saturated salt solution of CH_3_COOK was used to control the relative humidity in the chamber at 23%. Samples were placed on a rack above the salt solution and the area of exposure was kept as large as possible in order to allow free diffusion of water. The chamber was maintained at 45°C and ventilated to increase mass transfer for 1 h. The conservation time before experiments lasted about 24 h, and the samples were kept in the chamber at 23% relative humidity at 25°C.

### Yeast Survival

The yeast survival was investigated via a cell cultivability test using the Colony-Forming Unit (CFU) method. For dehydrated yeast, the cells were rapidly rehydrated in an acetate buffer. Decimal dilutions of samples (hydrated and dehydrated/rehydrated cells) were plated in YPDA medium containing 20 g/L Glucose, 20 g/L Peptone, 10 g/L Yeast extract, and 16 g/L Agar. CFUs were counted after incubation for 36 h at 30°C. All of the experiments were carried out in triplicate from independent pre-cultures and cultures.

### Synchrotron FTIR Microspectroscopy

**FIGURE 1:**
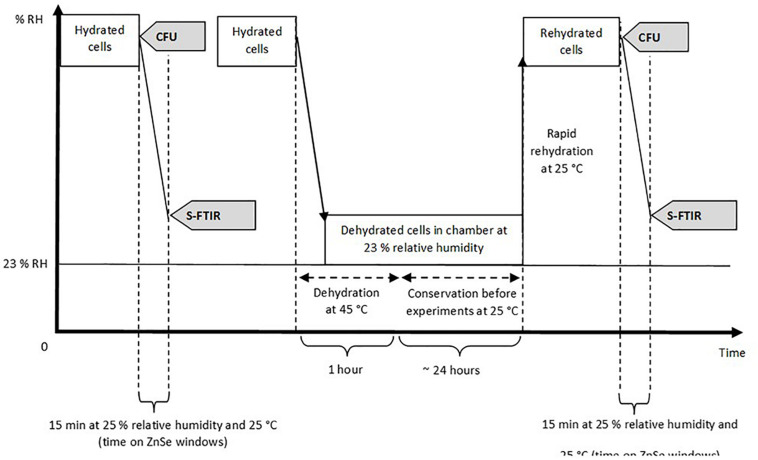
Experimental procedure used and analysis performed on the samples. Hydrated and rehydrated cells represent both encapsulated and non-encapsulated cells before and after the dehydration process at 45°C. CFU (Colony-Forming Unit) was used to estimate cell cultivability. S-FTIR (Synchrotron FTIR microspectroscopy) was used to assess the biochemical composition of yeast cells. Dehydration and rehydration kinetics of *Saccharomyces cerevisiae* BY4742 during air-drying were illustrated in a previous study (Nguyen et al., 2017).

Synchrotron infrared microspectroscopy was performed at the SMIS beamline at the SOLEIL Synchrotron Facility (Saint-Aubin, France). The beamline is equipped with a Thermo Scientific Continuum infrared microscope coupled with a Thermo Scientific Nicolet 5700 spectrometer. The S-FTIR measurement was performed in transmission mode using ZnSe windows with a similar protocol as previously described by Nguyen et al. (2017). As yeast cells were around 5 μm in diameter, the aperture was adjusted at 5×5 μm^2^ in order to collect the infrared signal at single cell level. The sample preparation and the experimental steps, reported in Figure 1, followed similar procedures as those described in our previous paper (Nguyen et al., 2017).

**FIGURE 2:**
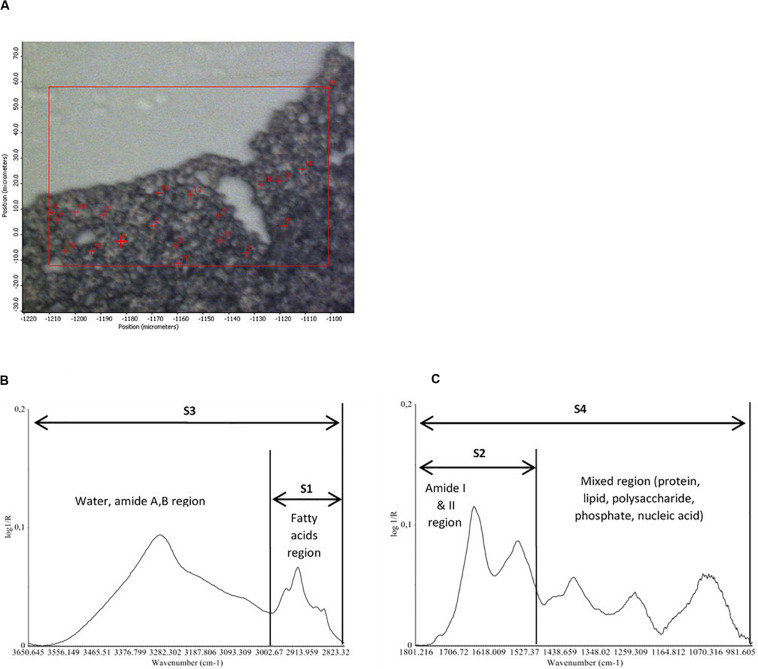
Map of 20 randomly selected individual cells for infrared spectra acquisition **(A)**. Representative corrected FTIR spectra of an individual cell of *Saccharomyces cerevisae* strain (non-encapsulated cell before the dehydration process) in the four spectral regions (S1, S2, S3, and S4) used for data analysis **(B,C)**.

For infrared spectra acquisition, 2 μL of hydrated cells (encapsulated and non-encapsulated cells) were directly deposited on the ZnSe window. Dehydrated cells (encapsulated and non-encapsulated), which were previously dehydrated in a dedicated chamber at 23% relative humidity, were rapidly rehydrated in an acetate buffer and then 2 μL of dehydrated/rehydrated cells were deposited on the ZnSe window. Samples remained on the ZnSe window at room temperature for 15 min before spectral acquisition. The room temperature was 25°C and relative humidity was 25% (measured by Radiospare RS1364 Humidity-Temperature Meter). Individual spectra were acquired at 4 cm^–1^ spectral resolution, with 256 scans encompassing the mid-infrared region from 4000 to 800 cm^–1^. The high spatial resolution was obtained without compromising the quality of the infrared signal thanks to the synchrotron infrared radiation. For each condition tested, analyses were carried out on around 20 to 25 individual cells via a map (Figure 2) using the OMNIC software (Thermo Fisher Scientific, Madison, WI, United States).

### Statistical Analysis

The analysis of the S-FTIR data was performed using the Unscrambler software (version 10.2, CAMO Process AS, Oslo, Norway). The analysis of the S-FTIR data was performed over four spectral regions (Figure 2):

(i) S1 (3010–2800 cm^–1^): the lipid region, exhibiting the C-H stretching vibration of the CH_3_ and CH_2_ functional groups. The region is dominated by the spectral characteristics of fatty acid chains of the various phospholipid membranes and by some amino side-chain vibrations.
(ii) S2 (1800–1500 cm^–1^): the protein region dominated by the protein conformation-sensitive amide I and amide II bands.
(iii) S3 (3650–2800 cm^–1^): this large region exhibits the O-H stretching vibrations in carbohydrates, the stretching vibrations of N-H from proteins, and C-H stretching vibrations of methyl and methylene groups (S1 region).
(iv) S4 (1800–950 cm^–1^): the large S4 region is dominated by the protein amide bands (S2 region), by the mannans and glucans C-O-H and C-O-C vibrations, the P = O from phosphates in nucleic acids, and the C-H bending modes from lipids.

The raw spectra were pretreated as previously described (Passot et al., 2015). The raw spectra were first baseline-corrected by subtracting a linear baseline and then normalized using the Unit Vector Normalization of the Unscrambler software. In order to investigate membrane fluidity, the second derivatives of spectra obtained in the spectral region from 2900–2830 cm^–1^ (exhibiting the symmetric of C-H stretching vibration of methyl and methylene groups) were calculated (Savitsky–Golay, order 3, 7-point smoothing factor), followed by normalization using unit vector normalization.

After pre-processing, the data were analyzed by the unsupervised principal component analysis (PCA) method so that the natural variation pattern in the data could be studied. The PCA was carried out on mean centered data using the singular value decomposition algorithm and leverage validation method. 7 principal components were computed and the optimal number of components was found to be 3 or 4 in all analyses. Outliers were eliminated in a first round of PCA using the 95% confidence Hotelling T2 ellipse.

The score plots show the projection of each spectrum onto the space defined by the corresponding principal components. The loading plots of principal components relate the behavior of the score plots to the spectral modifications in the sample such as shifts in band position, increase or decrease in absorption band intensities. These variations can, in turn, be related to biochemical changes within the individual groups.

## Results and Discussion

### Yeast Survival and Induced Biochemical Modifications

*Saccharomyces cerevisiae* cells encapsulated or non-encapsulated were dehydrated at 45°C. Yeast survival was investigated by estimating cell cultivability. The cell cultivability of encapsulated and non-encapsulated yeast was evaluated before and after dehydration at 45°C (Table 1). Results exposed in Table 1 indicate statistical contrasts between samples at the 95% confidence level. Before dehydration at 45°C, no significant differences (*p* < 0.05) in the logarithm of CFU per mL between encapsulated and non-encapsulated cells indicated that encapsulation conditions did not affect yeast cultivability. Authors have previously demonstrated that *S. cerevisiae* cells preserved their metabolic activities and were able to divide after encapsulation process using LbL method (Diaspro et al., 2002; Drachuk et al., 2012).

**TABLE 1:** Cultivability counts (log CFU/mL) of *Saccharomyces cerevisiae* cells determined before and after dehydration.

	**Log CFU/mL**	
	**Before dehydration**	**After dehydration**
Non-encapsulated cells	7.32 ± 0.03^a^	3.96 ± 0.41^b^
Encapsulated cells	7.17 ± 0.06^a^	5.09 ± 0.35^c^

*Values with the same superscript letters are not significantly different at the 95% confidence level.*

The results showed a decrease in the survival of both encapsulated and non-encapsulated yeast cells after dehydration, indicating the destructive effect of this process. The decrease in cell survival due to dehydration has been historically demonstrated in the literature (Morgan et al., 2006; Dupont et al., 2011; Fu and Chen, 2011; Lemetais et al., 2012). However, the decrease in encapsulated cells (2 log) was lower than that in non-encapsulated cells (3 log). The shell therefore provided a protection to yeast during dehydration. The protective effect of encapsulation using the LbL method on yeast survival to dehydration caused by freezing and freeze-drying has been elsewhere demonstrated (Thomas et al., 2014; Nguyen et al., 2015). To the best of our knowledge, this is the first time that the protection afforded by biopolymer shells against dehydration in air-drying at 45°C has been reported in yeast.

The presence of biopolymers on yeast surface could induce biochemical modifications of cells. Consequently, encapsulated and non-encapsulated yeast cells were analyzed by S-FITR in order to identify biochemical differences. The biochemical characterization of individual cells was expected to help to better understand the higher survival of encapsulated compared to non-encapsulated yeast after a dehydration process. In our previous study, it was demonstrated that the average shell thickness of three layers (βlg/Alg/βlg) was approximately 140–190 nm (Nguyen et al., 2015).

**FIGURE 3:**
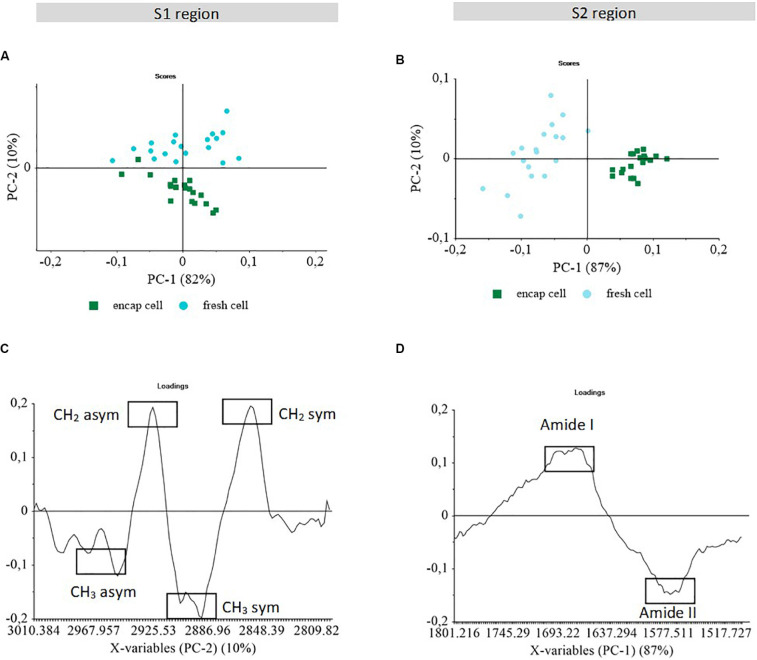
Principal component analysis of S-FTIR spectra obtained for encapsulated and non-encapsulated fresh cells in two spectral regions S1 and S2. PCA score plots **(A, B)**; and PCA loading plots **(C, D)** of PC1 vs. PC2. The score plots show the discrimination between samples. The loading plots indicate the spectral variance within and between samples.

Principal component analysis was used to identify specific regions of the S-FTIR spectra that contribute to the discrimination of the fresh yeast cells (both encapsulated and non-encapsulated). PCA was performed in all the spectral regions between 4000 and 950 cm^–1^). Only regions S1 (3010–2800 cm^–1^) and S2 (1800–1500 cm^–1^) of the S-FTIR spectra, involving information about lipids and proteins, contributed to the discrimination of the fresh yeast cells and were further studied. The first component (PC1) vs. the second component (PC2) score plots and the loading plots are presented in Figure 3.

The S1 region (3010–2800 cm^–1^) is dominated by the signal of the C-H stretching vibrations of CH_3_ and CH_2_ functional groups, corresponding to the fatty acid chains of the various membrane phospholipids and to some amino side-chain vibrations from proteins (Burattini et al., 2008; Passot et al., 2015). Although PC1 captured the highest spectral variability (82%), it appeared to be related to the cell dispersion within each sample (Figure 3A). This could be ascribed to differences in membrane composition of individual yeast cells.

Although the most important chemical heterogeneity within yeast populations was not related to encapsulation in this spectral region, the PCA score plots showed a near perfect cluster separation according to encapsulation along PC2 axis using 10% of spectral variance.

Non-encapsulated cells (fresh cell) clustered on the positive part, whereas encapsulated cells (encap cell) clustered on the negative part of the PC2 axis (Figure 3A). In Figure 3C, positive peaks in the loading plot of PC2 corresponded to spectral features associated with the non-encapsulated cell population and appeared to be specifically correlated to the C-H stretching vibration of the CH_2_ methylene group (2925 and 2850 cm^–1^). The encapsulated cells were characterized by CH_3_ methyl groups (2960 and 2890 cm^–1^). It indicated that the CH_2_:CH_3_ ratio was different between these two populations. The higher methyl groups contribution observed in encapsulated yeast could be due to the presence of β-lactoglobulin in the microorganized shell although the presence of alginate should increase the contribution of methylene group.

Furthermore, another hypothesis is that the change of CH_2_:CH_3_ ratio could be the consequence of changes in membrane composition, in particular in lipid composition. Higher contribution of CH_3_ bands could be related to higher number of short-chain fatty acids. In contrast, higher contribution of CH_2_ bands could reflect the presence of longer and/or more saturated acyl chain (Ami et al., 2009; Saulou et al., 2013). Such variation of the CH_2_:CH_3_ ratio could be related to the encapsulation process by generating acid stress and inducing change in membrane fluidity through membrane adaptation by the means of biochemical modifications. Yeast response to dehydration could depend on the length and saturation of fatty acid chains. In the study carried out by Passot et al. (2015), the authors reported that bacteria cells grown in a rich medium presented a higher content in CH_3_ groups assigned to lipid chains and a higher survival after dehydration by freezing. In the present work, a similar trend was found in terms of the relationship between higher number of short-chain fatty acids and higher survival after dehydration, as observed in the case of encapsulated yeast. Therefore, the membrane adaptation induced by the multilayer shells could led to a protective effect on yeast during dehydration process.

In the S2 region (1800–1500 cm^–1^), infrared spectral absorption exhibits the C = O stretching of amide I, C-N stretching and N-H bending of amide II. According to the PCA score plot, PC1 capturing 87% of spectral variability separated encapsulated cell (encap cell) from non-encapsulated cell (fresh cell) clusters (Figure 3B). Other principal components did not separate the two populations and carried information on the variability within the clusters, regardless of population considered. The most important chemical heterogeneity within yeast populations in this spectral region was therefore related to encapsulation. When considering PC1, the positive peaks of the amide I protein range were associated with non-encapsulated cell clusters, while the encapsulated cell population was characterized by the negative peaks of the amide II protein range (Figure 3D). This result evidenced a change in the amide I/amide II ratio between the two cell populations, thus suggesting that the secondary structure of protein in encapsulated cells was different from the secondary structure of protein in non-encapsulated cells. Different hypotheses can be put forward for explaining this result: (i) protein structure changes in existing proteins; (ii) expression of new proteins; (iii) proteins of the shell; or (iv) different dehydration kinetics when preparing the cells for FTIR. Furthermore, by analyzing second derivative of S-FTIR spectra in this specific region (data not shown) the denaturation of proteins was not observed following encapsulation. This result was also consistent with our previous work (Nguyen et al., 2015), showing that the electrostatic complexation between β-lactoglobulin and alginate only did not lead to modification in the secondary structure of protein. Thus, the amide band changes differentiating encapsulated and non-encapsulated cells, could probably be related to the changes in the CH3:CH2 ratio in the S1 region indicating the presence of β-lactoglobulin in the microorganized shell or the modification of cell protein content.

### Dehydration Induced Biochemical Modification in Yeast Cells

**FIGURE 4:**
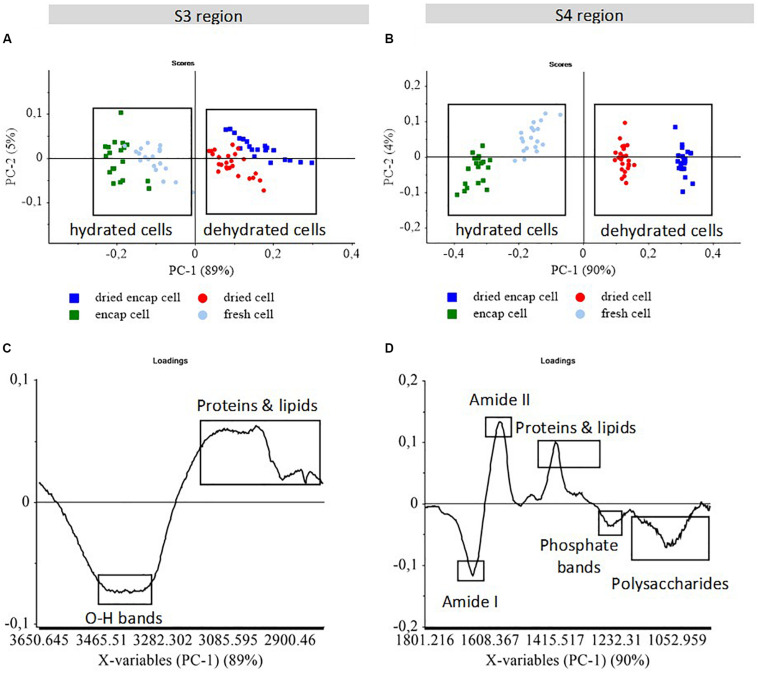
PCA score plots **(A,B)** of S-FTIR spectra of encapsulated and non-encapsulated yeast before and after dehydration in the two regions S3 and S4. PCA score plots **(A,B)**; and PCA loading plots **(C,D)** of PC1 vs. PC2. The score plots show the discrimination between samples. The loading plots indicate the spectral variance within and between samples. The boxes were added to help separating the clusters of high temperature dried cells and low temperature fresh cells.

The infrared spectra of individual encapsulated and non-encapsulated cells before (encapsulated cell and fresh cell) and after (dried encapsulated cell and dried cell) dehydration were monitored in two large regions, S3 (3650–2800 cm^–1^) and S4 (1800–1500 cm^–1^), which contained information on the water/biomass ratio and on biomolecules, respectively. These results are reported in Figure 4.

The S3 region (3650–2800 cm^–1^) includes the contribution of O-H stretching in carbohydrates around 3500–3400 cm^–1^, the N-H stretching in proteins at 3300 cm^–1^, the amide II overtone around 3100 cm^–1^, the absorptions mainly assigned to CH_3_ and CH_2_ functional groups (asymmetric vibrations at 2960 cm^–1^ and 2925 cm^–1^; symmetric vibrations at 2870 cm^–1^, and 2855 cm^–1^; Burattini et al., 2008). In Figure 4A, the score plot showed the spectral variability along the PC1 axis within the groups. Dried yeast cell populations were separated from fresh yeast cell populations by PC1 with 89% of the spectral variance. Thus, PC1 revealed the effect of dehydration, regardless of whether the cells were encapsulated or not. According to the PC1 loading plot (Figure 4C), the negative peak at 3400 cm^–1^ associated with O-H stretching vibration corresponded to the higher water content in the cell populations before dehydration that clustered on the negative part of the PC1 axis. The reduced O-H vibration absorption was thus clearly related to the decreased water content in yeast, resulting in osmotic stress which affected cell cultivability negatively (Gervais et al., 1992; Beney et al., 2000; Lemetais et al., 2012). The higher contribution of proteins and fatty acid bands observed in dried cells could be due to the water/biomass ratios or simply due to the spectral normalization procedure.

The S4 region (1800–950 cm^–1^) carries the absorption peaks from proteins, lipids, nucleic acids, and cell wall polysaccharides (mannans and glucans; Burattini et al., 2008; Gautier et al., 2013; Passot et al., 2015). The PCA score plot (Figure 4B) showed a clear separation between fresh cell and dried cell populations along PC1 with 90% of the spectral variance. The fresh cells were found on the negative part while the dried cells were positioned on the positive part of the first principal component. Therefore, the spectral variability monitored by PC1 revealed the effect of dehydration in this spectral region. Encapsulated and non-encapsulated cells were also separated along PC1 axis showing that there was more organic matter in the encapsulated cells than in the non-encapsulated cells, even when dried.

**FIGURE 5:**
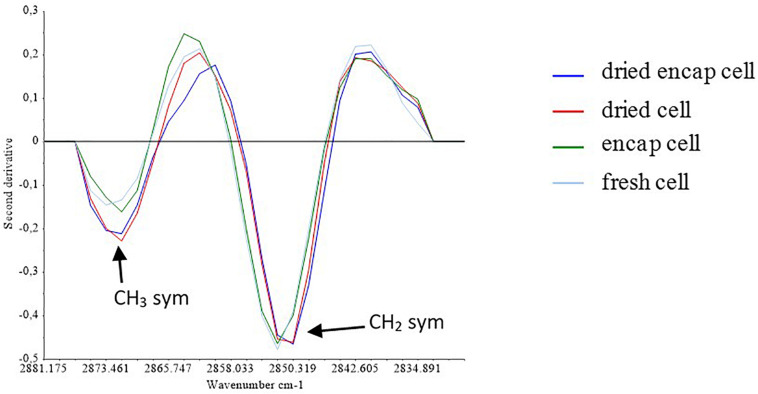
Second derivate of the spectra in the S1 region.

**FIGURE 6:**
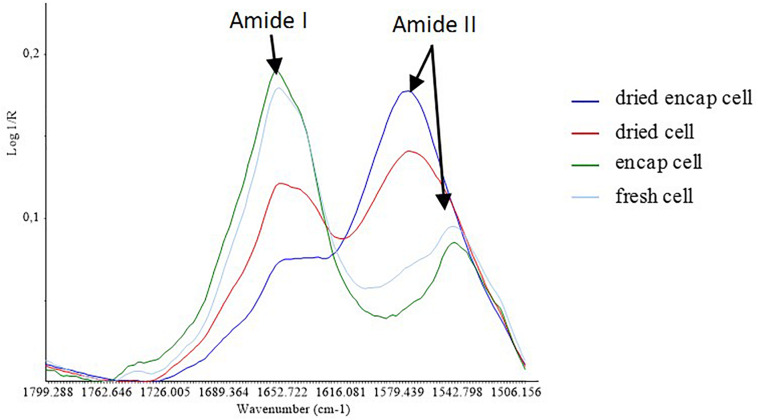
Average spectra of the samples in the S2 region.

According to the PC1 loading plot (Figure 4D), the negative peaks of amide I (1660 cm^–1^), polysaccharide (1200–900 cm^–1^), and phosphate (1247 cm^–1^) bands appeared to be associated with the fresh cells, while the dried cells were characterized by a higher contribution of amide II (1575 cm^–1^) and proteins and lipids (1500–1300 cm^–1^) bands. The discrimination between fresh and dried cells could therefore be ascribed to differences in secondary protein structure, RNA and cell wall composition. Ishida and Griffith reported a strong change in the amide I/amide II ratio for β-lactoglobulin in solution compared to dehydrated films (Ishida and Griffiths, 1993). This could explain the strong amide I/amide II changes at 1660 cm^–1^ and 1575 cm^–1^ in PC1 loadings. Differences in the intensity of proteins, lipids, and polysaccharides bands between hydrated and dehydrated cells could be due to the amount of water in samples.

principal component analysis results of two large regions S3 and S4 showed clearly differences in yeast response to dehydration. In order to observe more precisely the effect of the dehydration process on yeast composition, we analyzed the mean S-FTIR spectra of each population in the specific regions S1 and S2 containing information for lipids and proteins. The results were reported in Figures 5, 6.

Figure 5 presented the second derivative of mean spectra for encapsulated and non-encapsulated cells before and after the dehydration process at 45°C in the specific region (from 2900 to 2830 cm^–1^) containing the contribution of the symmetric C-H stretching vibration of the CH_2_ and CH_3_ groups. After dehydration, the intensity of the CH_3_ symmetric stretching band at 2875 cm^–1^ increased relative to the methylene band. This indicates that the ratio of the number of methylene groups to that of methyl groups decreased in dried samples, as compared to hydrated samples (both encapsulated and non-encapsulated cells). The decrease in number of methylene groups could correspond to the reduction of the number of long-chain fatty acids, which could originate from the degradation of lipid by hydrolysis or oxidation (Ami et al., 2009; Fuchs et al., 2011). This suggested changes in the lipid membrane during dehydration process. Burattini et al. (2008) reported the reduction of the ν_sym_(CH_2_)/ν_sym_(CH_3_) intensity ratio, which can be attributed to the degradation of fatty acid chains by β-oxidation, in their study of autolysis in cells of the wine yeast *S. cerevisiae* (Burattini et al., 2008). Moreover, the difference in the ν_sym_(CH2)/ν_sym_(CH3) intensity ratio of non-encapsulated cells before and after dehydration at 45°C seemed higher than that of encapsulated cells (∼30% higher). It means that the length of the fatty acid chains of non-encapsulated cells were more negatively affected by the dehydration process at 45°C than that of encapsulated cells. The presence of micro-organized shells can therefore better preserve the lipid composition of yeast during dehydration.

Furthermore, dehydration can induce change in membrane fluidity and can be related to cell death. Some authors have reported that cell membranes became more rigid for water activity values from 0.88 to 0.37 during yeast dehydration at constant temperature (Simonin et al., 2008). The band position of symmetric C-H stretching vibration corresponding to CH_2_ groups was reported as indicator of membrane fluidity (Alvarez-Ordóñez et al., 2010). In the Figure 5, a shift of the CH_2_ bands to a lower wavenumber is observed following dehydration, regardless of whether the cells were encapsulated or not. This could be associated with reduction of membrane fluidity. Passot et al. (2015) similarly found that freezing resulted in a downshift of the symmetric C-H vibration of the CH_2_ methylene groups. Another study reported that the downshift of the symmetric CH_2_ stretching peak associated with cell death (Saulou et al., 2013). In the present work, the presence of micro-organized shells could not preserve membrane fluidity of yeast during dehydration. Furthermore, yeast survival has been reported to depend on the dehydration and rehydration kinetics. Authors reported that higher survival was observed when the dehydration/rehydration process was progressive (Lemetais et al., 2012). The presence of micro-organized shells on yeast could induce a protective effect by a physical effect. The shell could be a barrier that reduced the kinetics of water transfer during dehydration/rehydration cycle, leading to higher cell survival.

Focusing on the amide I and amide II protein region (Figure 6), the spectral profile of yeast after the dehydration process at 45°C was different from that of yeast before treatment. After dehydration, the intensity of amide I bands (∼1660 cm^–1^) decreased in both encapsulated and non-encapsulated cells. The amide I vibration is hardly affected by the nature of the side chain. It depends instead on the secondary structure of the backbone and presents strong bands at 1653 cm^–1^ and 1633 cm^–1^. These bands are described in the literature as being features of α-helix and β-sheet structures (Oberg et al., 2004). No significant shift was observed for these bands suggesting limited effect in ordered secondary structure. Furthermore, our results showed the appearance of a peak at 1575 cm^–1^ after the dehydration process at high temperature in both encapsulated and non-encapsulated cells. Oberg et al. (2004) reported that amide II provides significant information and could be used alone for the prediction of protein secondary structure. The amide II bands are recognized in the main peak at 1550 cm^–1^ (Barth, 2007; Cavagna et al., 2010). However, the data regions from 1590 to 1500 cm^–1^ (Oberg et al., 2004; Movasaghi et al., 2008) or from 1480 to 1575 cm^–1^ (Kong and Yu, 2007) have been described associated to amide II bands. The appearance of the peak at 1575 cm^–1^ might therefore be considered as a shift from the peak at 1550 cm^–1^ to the new one, linked to modifications in protein conformation. However, other chemical modifications cannot be excluded. Indeed, Palacios and Juárez-López (2004) and Otero et al. (2014), have assessed by IR spectroscopy that peak at 1575 cm^–1^ corresponded to Fe(III) carboxylate and Ca(II) carboxylate, respectively, suggesting that such metallo-complexes could be formed during dehydration in our experimental conditions.

## Conclusion

This study provided evidence for the application of the LbL encapsulation method on *S. cerevisiae* in order to protect yeast cells against dehydration at high temperature and highlighted the mechanism impact of this process on yeast cells. Moreover, protective mechanism of microorganized shells on yeast responses was proposed. The lower CH_2_:CH_3_ ratio observed in lipid spectral region could be ascribed to the encapsulation process without clear differentiation between the presence of organic matter at the cell interface or modification of membrane chemical composition. During dehydration, encapsulated yeast presented a higher survival and a higher methyl group contribution. Alternatively to direct chemical modifications of the cell structure, the presence of hydrocolloids at the cell interface could be a barrier limiting stress due to water transfer upon dehydration-rehydration cycle. The coupled study of biochemical and physiological responses of cells following different kinetics of dehydration/rehydration would confirm the role of microorganized shells on yeast resistance to dehydration.

## Data Availability Statement

The datasets generated for this study are available on request to the corresponding author.

## Author Contributions

TN, RS, and FH contributed to the conception and design of the study. TN, SG, CP, SP, CS, FF, RS, and FH did all the experiments at Synchrotron Soleil. TN wrote the first draft of the manuscript. All authors contributed to manuscript revision, read and approved the submitted version.

## Conflict of Interest

The authors declare that the research was conducted in the absence of any commercial or financial relationships that could be construed as a potential conflict of interest.
